# Highlight: Recracking the Genetic Code

**DOI:** 10.1093/gbe/evz211

**Published:** 2019-10-25

**Authors:** Casey McGrath

Crack open a biology textbook and you will find a standard icon of molecular biology: the table summarizing the standard genetic code. This refers to the set of rules by which the cell “decodes” the information contained in DNA and “translates” it into the amino acids that make up proteins. For example, in virtually all organisms, the codon (3-letter DNA sequence) “AGA” tells the translation machinery to add the amino acid asparagine. Although a few deviations in the genetic code used by various organisms are known, research recently published in *Molecular Biology and Evolution* and in this issue of *Genome Biology and Evolution* suggests that we may have only begun to scratch the surface on the number of variations present in the genetic codes of all living organisms.

In the April issue of *Molecular Biology and Evolution*, a group of researchers including Emmanuel Noutahi, Virginie Calderon, Mathieu Blanchette, Nadia El-Mabrouk, and Bernd Franz Lang from the Universite de Montreal, Institut de Recherches Cliniques de Montreal, and McGill University published an analysis of the mitochondrial genomes of 51 green algae and land plants (Noutahi et al. 2019). This analysis relied on a newly expanded version of the bioinformatics tool CoreTracker, which was previously developed by this group (Noutahi et al. 2017). CoreTracker identifies differences between a DNA sequence and the expected amino acid based on the amino acids often found at that position in closely related species. Using this tool, Noutahi and colleagues identified 14 new codon reassignments involving the replacement of one amino acid with another, the vast majority of which were found in a group of algae known as the Sphaeropleales. These algae have an unusual mitochondrial genome organization that appears to be intermediate between the larger, ancestral genomes and the compact, derived genomes of some of their relatives.

According to the authors, the field of genetic code evolution is being fueled by a rapid increase in genomics data (genomes plus respective transcriptomes). Because of this, “Comparative/evolutionary bioinformatics procedures such as CoreTracker are now in a position to not only predict deviations of the genetic code but also provide clues with regard to the underlying mechanism.” Indeed, in light of their results, the researchers proposed that the genetic code deviations in the Sphaeropleales mitochondria actually contributed to their unusual genome organization. Based on this theory, after the migration of some mitochondrial genes to the nuclear genome during the genome reduction process, “UCA” (normally encoding the amino acid serine) was reassigned to a termination codon. This would have made it impossible for additional mitochondrial genes to be transferred to the nucleus, resulting in a mitochondrial genome that was intermediate in terms of size.

Before the publication of the article by Noutahi et al., researchers David Žihala and Marek Eliáš from the University of Ostrava had also independently discovered the large number of changes to the genetic code in the Sphaeropleales. Following the purely coincidental discovery of novel genetic codes in several protists by Eliáš’s research group, Žihala and Eliáš were motivated to “embark on a systematic screening to find possible additional cases of organisms with novel genetic code variants or previously missed departures from the standard genetic code.” Like Noutahi et al., their method involved the identification of discrepancies between DNA sequences and expected amino acids based on sequences present in related genomes, although they also performed a certain amount of manual curation. The analysis, published in the current issue of *Genome Biology and Evolution* (Žihala and Eliáš 2019), identified a few more codon reassignments in the Sphaeropleales, due to the fact that they included a broader sampling of this group. Otherwise, the results of the two studies were highly congruent, despite the somewhat different methods employed. In addition to the genetic code changes, Žihala and Eliáš also identified mutations in a mitochondrial release factor—a protein that recognizes termination codons—that, according to Eliáš, “may be linked to the intriguing ability of some sphaeroplealean mitochondria to terminate translation at codons that are normally read as coding for an amino acid. We thus offer the first specific hypothesis for the molecular underpinnings of this unusual ability.”

Overall, the findings of both studies highlight the need for a deeper awareness of genetic code discrepancies across the tree of life. Otherwise, use of an incorrect code when inferring protein sequences from DNA sequences could lead to inaccuracies in predicted protein sequences that are used for both phylogenetic and molecular biology analyses. Moreover, according to Noutahi and colleagues, “The two publications have been successful in predicting specific changes in the meaning of codons by using publicly available data, without biochemical experimentation, yet with high confidence.” However, they also note that both studies are strictly computational in nature and that “this type of ‘paper biochemistry’ has its limitations. Only evolutionarily well-established instances of codon evolution may be inferred (i.e., not including cases of initial or incomplete stages), and although changes to the tRNA repertoire, structure, and specificity may be inferred to some degree, biochemical confirmation is critically required.” This limitation was also pointed out by Eliáš, who noted that his group plans to employ proteomics methods to verify some of their bioinformatic predictions concerning the various presumably reassigned codons. “Unfortunately,” continues Eliáš, “nonstandard genetic codes are generally found in organisms that are difficult to study by direct biochemical or genetic approaches, so it remains a challenge to get a deeper understanding of the molecular mechanisms behind the observed changes in codon meaning.”

Future investigations will almost certainly uncover additional, as-yet-unidentified changes in the genetic code in various organisms. Indeed, notes Eliáš, “We are also analyzing some exciting new cases of genetic code modification in nuclear genomes of certain obscure protists that were uncovered by our survey of publicly available sequence data.” Furthermore, the authors of the Noutahi et al. study point out that, given “how rapidly this field is evolving, due to an ever increasing number of reports on deviations from the standard genetic code—particularly, in eukaryotes and their organelles… the journey to understanding codon evolution with all of its mechanistic implications has just begun.” 
